# Relationship between Serum miR-106 and MYL4 Levels and the Prevalence, Risk Stratification, and Prognosis of Atrial Fibrillation

**DOI:** 10.1155/2022/1069866

**Published:** 2022-07-14

**Authors:** Yanfeng Liu, Haiwen Zhou, Yu Tao, Zhicheng Xu, Hengli Lai

**Affiliations:** Department of Internal Medicine-Cardiovascular, Jiangxi Provincial People's Hospital, The First Affiliated Hospital of Nanchang Medical College, Nanchang 330000, Jiangxi Province, China

## Abstract

**Objective:**

To analyze the predictive value of serum microRNA-106 (miRNA-106), miR-106, and myosin light chain 4 (MYL4) levels on the prevalence of atrial fibrillation and to explore the relationship between serum miR-106 and MYL4 and the risk stratification and prognosis of atrial fibrillation, thereby providing basis for them to become clinical targets for the treatment of atrial fibrillation in the future.

**Methods:**

300 patients with atrial fibrillation treated in our hospital from May 2017 to March 2019 were selected as the atrial fibrillation group, and 300 healthy people who came to our hospital for physical examination in the same period were selected as the control group. The general data of the subjects in the two groups were collected. The serum miR-106 level of the subjects in the two groups was detected by fluorescence quantitative polymerase chain reaction (PCR), and the level of MYL4 was detected by enzyme-linked immunosorbent assay (ELISA). The expression of miR-106 and MYL4 in the myocardium was observed by immunohistochemistry. The relationship between the levels of serum miR-106 and MYL4 and the prevalence of atrial fibrillation and the score of atrial fibrillation thromboembolism risk stratification scoring system (cha2ds2) was compared between the two groups. The relationship between serum level of miR-106 and prognosis of patients with atrial fibrillation was analyzed.

**Results:**

The systolic blood pressure, diastolic blood pressure, total cholesterol (TC), triglyceride (TG), low-density lipoprotein cholesterol (LDL-C), and left anterior descending artery (LAD) in the atrial fibrillation group were significantly higher than those in the control group, while HDL-C and left ventricular ejection fraction (LVEF) were significantly lower than those in the control group (*P* < 0.01). The level of serum miR-106 in patients with atrial fibrillation was significantly higher than that in the control group, whereas the level of MYL4 was significantly lower than that in the control group (*P* < 0.01). miR-106 was mainly localized in the cytoplasm, and the positive expression rate of miR-106 was 71.43% (81/115) in patients with atrial fibrillation and 21.74% (25/115) in patients with sinus rhythm. MYL4 was mainly located in the cell membrane and the positive expression rate of MYL4 was 24.35% (28/115) in patients with atrial fibrillation and 64.35% (74/115) in patients with sinus rhythm. With the increase of the severity of atrial fibrillation, the level of serum miR-106 gradually increased and the level of MYL4 gradually decreased, which were statistically significant compared with the control group (*P* < 0.05). With the increase of miR-106 level and the decrease of MYL4 level, the prevalence of atrial fibrillation gradually increased. With the increase of cha2ds2 score, the level of serum miR-106 increased and the level of MYL4 decreased. The survival rate of patients with miR − 106 ≤ 1.96 was significantly higher than that of patients with miR − 106 > 1.96. The survival rate of patients with MYL4 ≥ 0.24 was significantly higher than that of patients with MYL4 < 0.24. At the same time, TC and LDL-C were included in the analysis. The results showed that the survival rate of patients with TC ≤ 4.5 mmol/L was significantly higher than that of patients with TC > 4.5 mmol/L, and that of patients with LDL-C ≤ 2.6 mmol/L was significantly higher than that of patients with LDL-C > 2.6 mmol/L.

**Conclusion:**

Serum miR-106 and MYL4 levels are closely related to the prevalence of atrial fibrillation, which can reflect the risk of thromboembolism in patients with atrial fibrillation and can be used as a biological indicator to predict the prognosis of patients with atrial fibrillation.

## 1. Introduction

Atrial fibrillation (AF) is a persistent arrhythmia, accounting for about 1/3 of all patients with arrhythmia, with high incidence, disability rate, and great harm, and has been recognized as an independent risk factor for stroke [1]. At present, its pathophysiological mechanism has not been fully elucidated. Except for traditional risk factors, genetic susceptibility and epigenetic modification are also considered to be important endogenous factors in the development of AF [2].

In recent years, the regulation of microRNAS (miRNAs) in AF has become a research hotspot. Relevant data show that miRNAs may regulate the occurrence and maintenance of AF through regulating cardiac remodeling, ion channels, and energy metabolism, suggesting that miRNAs are expected to bring new directions for the therapy of AF [3]. Although the upregulation of miR-106b has been demonstrated to inhibit endothelial cell angiogenesis and participate in the pathogenesis of atherosclerosis [4], there are few reports on the mechanism of miR-106 in atrial fibrillation and other cardiomyopathy. In recent years, studies have suggested that myosin light chain 4 (MYL4) is associated with atrial development and atrial dysfunction, and it has been found that atrial cardiomyopathy is associated with MYL4 gene dislocation mutation, which is the pathogenic gene leading to atrial quiescence and AF [5]. AF and stroke are the clinical manifestations and adverse consequences of atrial cardiomyopathy. MYL4-related atrial cardiomyopathy is a typical example of electrophysiological abnormalities caused by abnormal atrial mechanical systolic function. MYL4 not only maintains sarcomere structure but also regulates cardiac diastolic and systolic function through ion channels or energy metabolism pathways [6]. Although previous studies have revealed that MYL4 gene mutation is closely related to familial atrial fibrillation and familial atrial quiescence, no report has focused on the relationship between serum MYL4 levels and atrial fibrillation. It is hoped that this study can provide the basis for serum MYL4 as a biomarker for the prevalence and risk stratification of atrial fibrillation and further explore the mechanism of miR-106/MYL4 in atrial fibrillation.

Therefore, in this study, by observing the changes of serum miR-106 and MYL4 levels in patients with AF, we analyzed the relationship between miR-106 and MYL4 and the prevalence, risk stratification, and prognosis of AF and provided theoretical and experimental basis for the diagnosis and treatment of AF.

## 2. Materials and Methods

### 2.1. General Data

**Figure 1:**
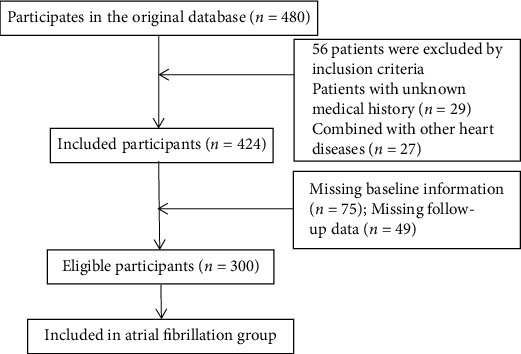
Flowchart of general data selection.

A total of 300 patients with AF from May 2017 to March 2019 were pitched as the research subjects. This study was approved by the ethics committee of our hospital. The following are the inclusion criteria: (1) all patients were confirmed by 12-lead ECG and 24-hour Holter monitoring with ectopic AF; (2) all patients with AF had AF duration > 1 year; (3) the clinical medical records were complete and cooperated with the research; and (4) all research subjects signed the informed consent. The following are the exclusion criteria: (1) patients with definite familial AF; (2) patients with unknown history of AF; (3) patients undergoing cardiac surgery; (4) patients with concomitant heart valve disease, congenital heart disease, pericarditis, and other heart diseases; (5) patients complicated with severe hepatic and renal insufficiency; and (6) patients with autoimmune diseases and connective tissue diseases. The 300 patients with AF included 155 males and 145 females, aged 31-88 years, with an average of 59.21 ± 12.12 years, 88 cases of cardiac function II, 189 cases of grade III, and 23 cases of grade IV. According to the frequency, duration, and treatment of AF, they were divided into 124 cases of paroxysmal AF, 113 cases of persistent AF, 42 cases of long-term persistent AF, and 21 cases of permanent AF. Another 300 healthy people (for physical examination in the same period) were chosen as the control group, including 150 males and 150 females, aged 35-87 years, with an average of 58.50 ± 13.84 years. The flowchart of general data selection is shown in Figure 1.

### 2.2. Method

1. The general information is as follows: gender, age, heart rate, weight and height [body mass index (BMI)], history of smoking and drinking, and blood pressure (systolic and diastolic) in both groups were collected. In the AF group, fasting venous blood was taken in the morning on the second day after admission, and venous blood was taken in the control group during physical examination. An automatic biochemical analyzer was applied to investigate the contents of triglyceride (TG), low- and high-density lipoprotein cholesterol (LDL-C, HDL-C), and total cholesterol (TC). The left atrial diameter (LAD) and left ventricular ejection fraction (LVEF) of the two groups were obtained by echocardiography
2. For miR-106 detection, collect fasting peripheral blood from subjects, centrifuge at 1500 rpm for 10 minutes to collect serum, and store at -80°C. Serum total miRNA was extracted with miRNA serum kit (model DP503, Beijing Tiangen Biochemical Company), and the concentration and purity were determined by spectrophotometer, and fluorescence quantitative PCR was performed. Steps are as follows: take 1 *μ*g of total RNA, and perform reverse transcription reaction with reverse transcription (RT) kit to obtain cDNA. PCR reaction system (20 *μ*L system) is as follows: 1 *μ*L of cDNA, 1.5 *μ*L of 10× Taq Buffer (containing Mg^2+^), 0.2 *μ*L of 10 mmol/L dNTP, 0.2 *μ*L of enzyme, 0.3 *μ*L of upstream and downstream primers, and DEPC water to 20 *μ*L. Shake and mix well, centrifuge at high speed for 10 s, and then set up PCR thermal cycler for amplification. Amplification conditions were predenaturation at 95°C for 3 min; denaturation at 94°C for 30 s, annealing for 40 s, extension at 72°C for 1 min, and 35 cycles; and final extension at 72°C for 10 min, and the amplified products were electrophoresed in 1.5% agarose gel; scan and analyze the bands by the gel imaging analysis system
3. For MYL4 detection, fasting peripheral blood was collected from subjects, centrifuged at 1500 rpm for 10 min to collect serum, and stored at -80°C, and ELISA (Abcam, UK) was used to detect the content of MYL4 in peripheral blood. Briefly, the serum samples were taken out to equilibrate at room temperature. Then, 100 *μ*L of the PBS and samples was added into blank wells and sample wells, respectively, mixed well and incubated at 37°C for 40 min. After, wash the reaction plate at least 4-6 times with washing solution and dry fully; each well except for the blank wells was added with 50 *μ*L of distilled water and 50 *μ*L of primary antibody working solution, mixed in the reaction plate well, and incubated at 37°C for 20 min. Next, each well was washed for 4-6 times, added 100 *μ*L of enzyme-labeled antibody working solution, mixed in the reaction plate well, and incubated at 37°C for 10 min. Subsequently, following wash for 4-6 times, each well was added into 100 *μ*L of substrate working solution and reacted at 37°C in the dark for 15 min. Finally, 100 *μ*L of stop solution was added into each well and mixed for 30 min. The optical density (OD) was measured at 450 nm with an enzyme labeler
4. For the expression of miR-106 and MYL4 in myocardial tissue, 115 of 300 patients with AF received thoracotomy treatment, and right auricular atrial muscle specimens were collected from patients with sinus rhythm undergoing cardiac surgery with the same sample size. The expression of miR-106 in atrial myocardium was detected by in situ hybridization, and the expression of MYL4 in atrial myocardium was observed by immunohistochemical staining. Steps are as follows: specimens were fixed with 4% paraformaldehyde, embedded in conventional paraffin, sectioned, deparaffinized to water, and washed with PBS. Prepare 3% H_2_O_2_ in distilled water, seal at room temperature for 5-10 min, and wash with distilled water 3 times. It was placed in pH 6.0 citrate buffer and microwave antigen retrieval for 15 min and incubated with 10% donkey serum blocking solution. A 1 : 100 dilution of sheep anti-human miR-106, MYL4 antibody, and type I collagen antibody was added to the sections labeled with miR-106 and MYL4, respectively, and incubated overnight at 4°C. After washing with PBS, rabbit anti-goat IgG secondary antibody was appended and incubated at 37°C for 25 min. After washing with PBS, the reagent SABC was added dropwise and incubated at 20°C-30°C for 20 min. Then, conduct DAB color rendering, dehydration, transparency, sealing, and microscopic examination
5. AF thromboembolic risk stratification scoring system (CHA2DS2) is as follows: heart failure/left ventricular ejection fraction < 40%, 1 point; hypertension, 1 point; age > 75 years, 2 points; diabetes, 1 point; stroke/cerebral thrombosis, 2 points; 1 point for vascular disease; 1 point for age 65-74 years; and 1 point for women. They were divided into seven groups according to 0~6 points. Serum miR-106 and MYL4 levels were compared in each group

### 2.3. Statistical Method

All data in this study were measured using $\bar{x}\pm s$ and analyzed using the SPSS 20.0 software package with the normality test. The difference in two groups was compared by an independent sample *t* test when the data conformed to a normal distribution. The values in multiple groups were compared by one-way analysis of variance when the data did not conform to a normal distribution. The count data were expressed as *n* (%). The *χ*^2^ test or Fisher's exact probability method was used to compare the data between the two groups. Survival curves were drawn by the Kaplan-Meier method, and the survival rates were compared using the log-rank test. *P* < 0.05 was considered statistically significant.

## 3. Results

### 3.1. Comparison of General Data of Two Groups

The heart rate, systolic blood pressure, diastolic blood pressure, TC, TG, LDL-C, and LAD in the AF group were signally larger than those indicators in control, and HDL-C and LVEF were prominently lower than those in control. The differences were statistically significant (*P* < 0.01). See Table 1.

### 3.2. Comparison of Serum miR-106 and MYL4 Levels

The serum concentration of miR-106 in the AF group was observably higher than that in the control group, and the level of MYL4 was markedly fewer than that in the control group, with memorable significance (*P* < 0.01). See Table 2.

### 3.3. Expression of miR-106 and MYL4 in Right Atrial Appendage Atrial Myocardium

**Figure 2:**
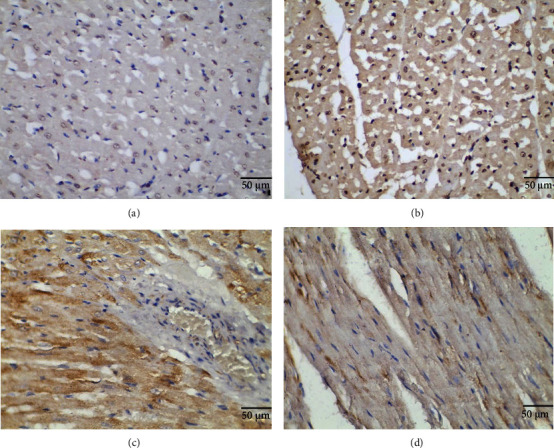
Expression of miR-106 and MYL4 in right atrial appendage atrial muscle tissue of patients with AF and sinus rhythm (×400). (a) miR-106 expression in AF myocardium; (b) miR-106 expression in sinus rhythm myocardium; (c) MYL4 expression in AF myocardium; (d) MYL4 expression in the sinus rhythm myocardium.

The observation results showed that miR-106 was mainly located in the cytoplasm, and the positive expression rate was 71.43% (81/115) in the myocardial tissue of patients with AF and 21.74% (25/115) in patients with sinus rhythm. MYL4 was mainly localized in the cell membrane, and the positive expression rate was 24.35% (28/115) in the myocardial tissue of patients with AF and 64.35% (74/115) in patients with sinus rhythm. See Figure 2.

### 3.4. Comparison of Serum miR-106 and MYL4 Levels in Patients with Different Types of AF

With the increase of the severity of AF, the serum miR-106 level showed a gradually elevating trend, and the MYL4 level showed a gradually descending trend, and the differences were significant compared with the control group (*P* < 0.05). See Table 3.

### 3.5. The Relationship between Serum miR-106 and MYL4 Levels and the Prevalence of AF

**Figure 3:**
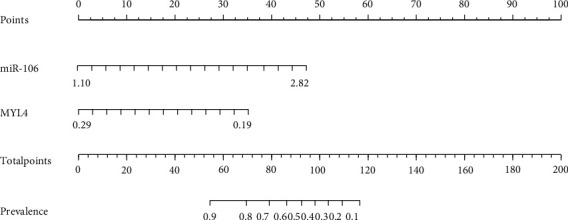
Nomogram of the relationship between miR-106, MYL4 and the prevalence of AF.

All the population (300 cases in the AF group and 300 cases in the control group) were divided into 4 groups according to the level of miR-106: Q1 < 1.10 (*n* = 150), 1.10 ≤ Q2 < 1.96 (*n* = 150), 1.96 ≤ Q3 < 2.82 (*n* = 151), and Q4 ≥ 2.82 (*n* = 149). The results demonstrated that with the increase of miR-106 level, the prevalence of AF was gradually ascending. Besides, the population was divided into 4 groups according to the level of MYL4: Q1 > 0.29 (*n* = 152), 0.29 ≥ Q2 > 0.24 (*n* = 149), 0.24 ≥ Q3 > 0.19 (*n* = 151), and Q4 ≤ 0.19 (*n* = 148). The results showed that with the decline of MYL4 level, the prevalence of AF was gradually increased (see Table 4 and Figure 3). Finally, after adjusting for age, blood pressure, blood lipids, smoking, drinking, and other clinical covariates, multivariate logistic regression analysis showed that the above differences were memorably significant (*P* < 0.05). See Table 5.

### 3.6. The Relationship between Serum miR-106 and MYL4 Levels and Risk Stratification of AF

With the increase of CHA2DS2 score, serum miR-106 level exhibited an increasing trend, while MYL4 level showed a decreasing trend. See Table 6.

### 3.7. The Relationship between Serum miR-106 and MYL4 Levels and the Prognosis of AF

**Figure 4:**
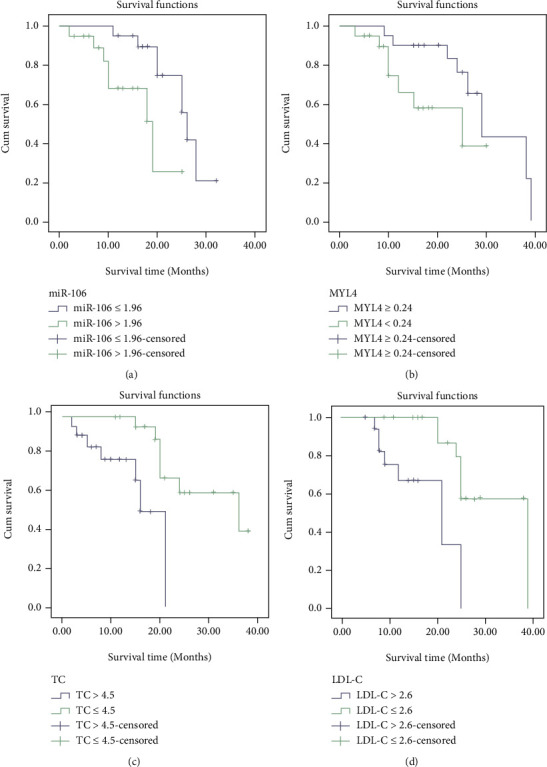
The relationship between serum miR-106 and MYL4 levels and the prognosis of AF. (a) Survival curve of patients with different miR-106 levels; (b) survival curve of patients with different MYL4 levels; (c) survival curve of patients with different TC levels; (d) survival curve of patients with different LDL-C levels.

The KM survival curve was applied to detect the levels of miR-106 and MYL4 and the prognosis of AF. The survival rate of patients with miR-106 ≤ 1.96 was memorably larger than that of patients with miR-106 > 1.96, and the survival rate of patients with MYL4 ≥ 0.24 was notably higher than that of MYL4 < 0.24. At the same time, TC and LDL-C were included in the analysis. The results demonstrated that the survival rate of patients with TC ≤ 4.5 mmol/L was memorably higher than that of patients with TC > 4.5 mmol/L, and the survival rate of patients with LDL-C ≤ 2.6 mmol/L was prominently greater than that of LDL-C patients with >2.6 mmol/L. See Table 7 and Figure 4.

## 4. Discussion

Due to the widespread aging of the population, the prevalence of AF is also increasing day by day, which greatly increases the risk of serious complications such as stroke, heart failure, and cardiomyopathy. It has become a crucial public health issue in developing countries. Therefore, it is crucial to clarify the pathogenesis of AF and to detect early and actively intervene. With the gradual deepening of the research on the electrophysiological mechanism of AF, atrial electrical remodeling is considered to be closely related to senile AF. With the prolongation of AF, the ultrastructure and extracellular mechanisms of atrial cardiomyocytes also change, which is called structural remodeling [7, 8]. Many previous studies have revealed the pathogenesis of AF, including the signaling pathway of Ca^2+^, and the remodeling caused by the inflammatory pathway, but its specific molecular biological mechanism has not been thoroughly studied. Moreover, it has been found that a variety of miRNAs were involved in the atrial structure and electrical remodeling process related to AF.

At present, the research on the relationship between miRNA and the heart has become one of the research hotspots. miRNAs belong to the class of noncoding small RNAs; they modulate the expression of their target genes by triggering mRNA degradation or protein translation repression. The mechanism of action of miRNA on AF is complex, and relevant data show that miRNA may regulate the occurrence and maintenance of AF by regulating myocardial remodeling, ion channels, and energy metabolism [9, 10]. Lu et al. found that the expressions of miR-223, miR-328, and miR-664 were observably higher in rheumatic heart disease complicated with AF and atrial pacing-induced canine AF models than those in the control group [11]. The mechanism of miR-106 in AF is less reported. Chiang et al. found that the total level of RyR2 in atrial tissues was increased (42%) in miR-106b and miR-25 knockout mice compared to the wild-type mice, but the distribution of RyR2 in the cytoplasm and endoplasmic reticulum remained normal. Calcium discharge frequency and total sarcoplasmic reticulum calcium leakage in atrial myocytes were promoted in the atrial myocytes of miR-106b and miR-25 knockout mice. Telemetric ECG recordings demonstrated that, compared with the wild-type immature mice, the miR-106b and miR-25 knockout mice displayed more frequent atrial ectopic beats and more sensitive to pacing-induced AF. In contrast, RyR2 blocker, K201, and blocked Ca^2+^ release from the sarcoplasmic reticulum and the increasing of sensitivity to AF in miR-106b and miR-25 knockout mice [12]. It may provide new clues for the elevation of intracellular calcium levels during the pathogenesis of AF.

In recent years, a number of studies have suggested that MYL4 is associated with atrial development and atrial dysfunction and found that MYL4 has a gene mutation, which is a pathogenic gene leading to atrial quiescence and AF [13]. MYL4 encodes a 197 amino acid myosin ELC, also known as atrial light chain, which is expressed in then cardiac and skeletal muscle during fetal life and specifically in atrial myocytes after birth. In congenital heart disease and ventricular cardiomyopathy, MYL4 expression in ventricular myocytes is reactivated, and its activation may be beneficial for improving cardiac systolic function. Research has indicated that the expression of MYL4 in ventricular myocytes is reactivated, and its activation may be beneficial to improve cardiac systolic function in congenital heart disease and ventricular cardiomyopathy. Loss of function of the MYL4 homolog cmlc1 by gene knockout in zebrafish results in the destruction of sarcomere structure and a severe decrease in cardiac contractile function [6, 14].

This study illustrated that the level of miR-106 in the AF group was markedly larger than that in the control, and the level of MYL4 was notably less than that control, suggesting that the increased level of serum miR-106 and the decreased level of MYL4 may be involved in the risk of AF, and is closely related to the severity and prevalence of AF. In addition, this study analyzed the relationship between serum miR-106 and MYL4 and the risk stratification of thromboembolism in AF and found that with the increase of CHA2DS2 score, the level of serum miR-106 was increased, and the level of MYL4 was decreased. It was suggested that serum miR-106 and MYL4 could reflect the risk of thromboembolism in patients with AF, providing guidance for clinical treatment. The changes of miR-106 and MYL4 indexes in the myocardial tissue of patients with AF may be affected by factors such as atrial overload, atrial systolic dysfunction, and decreased cardiac function; thus, these two indexes can be used as independent predictors of AF. The regulatory effect of miRNA on atrial remodeling in AF may be through the modulation of inward rectifying potassium channel protein expression and IKI current density, which may provide new prospects for the research and prevention of AF mechanism [15, 16]. Through KM survival curve analysis, it was found that the survival rate of patients with miR-106 ≤ 1.96 was dramatically higher than that of patients with miR-106 > 1.96, and the survival rate of patients with MYL4 ≥ 0.24 was observably higher than that of MYL4 < 0.24. It can be seen that serum miR-106 and MYL4 are closely related to the prognosis of patients with AF, which can be used as biomarkers to predict the prognosis of patients with AF.

In conclusion, the serum miR-106 content of patients with AF was prominently higher than that of healthy people and the MYL4 content was signally lower than that of healthy people, suggesting that abnormal serum miR-106 and MYL4 levels may predict the risk of AF. In addition, considering miRNA usually binds target genes to exert biological functions, this study speculates that the increase of miR-106 in serum of patients with AF may inhibit the expression of the downstream target gene MLY4, thereby promoting the susceptibility of AF and the changes of myocardial structure and ion channels. Whether there is a targeting relationship between miR-106 and MYL4 still needs to be verified by further clinical trials. In the future, more rigorous experiments will be required to explore from the aspects of genes, cells, and tissues to clarify the specific mechanism of the two in cardiomyocytes, thereby providing a basis for AF treatment targets.

## Figures and Tables

**Table 1:** Comparison of general data of the two groups of patients ($\bar{x}\pm s$) [*n* (%)].

General data	AF (*n* = 300)	Control (*n* = 300)	*χ* ^2^/*t*	*P*
Gender (male/female)	155/145	150/150	0.167	0.683
Age (year)	59.21 ± 12.12	58.50 ± 13.84	0.669	0.504
Heart rate (beats/min)	86.16 ± 7.12	70.44 ± 6.18	28.880	<0.001
BMI(kg/m^2^)	25.62 ± 3.74	25.19 ± 2.60	1.635	0.103
Systolic blood pressure (mmHg)	132.02 ± 11.49	125.58 ± 11.16	6.964	<0.001
Diastolic blood pressure (mmHg)	72.54 ± 10.85	67.25 ± 8.49	6.651	<0.001
Smoking history	62 (20.67)	60 (20.00)	0.041	0.839
Drinking history	57 (19.00)	72 (24.00)	2.222	0.136
TC (mmol/L)	3.82 ± 0.73	3.44 ± 0.79	6.119	<0.001
TG (mmol/L)	1.51 ± 0.49	1.16 ± 0.38	9.776	<0.001
HDL-C (mmol/L)	0.79 ± 0.32	0.93 ± 0.25	5.971	<0.001
LDL-C (mmol/L)	2.99 ± 0.42	2.44 ± 0.53	14.087	<0.001
LAD (mm)	45.46 ± 5.12	30.72 ± 2.16	45.943	<0.001
LVEF (%)	52.16 ± 4.57	62.25 ± 4.18	28.218	<0.001

**Table 2:** Comparison of serum miR-106 and MYL4 levels between the two groups ($\bar{x}\pm s$).

Groups	Cases	miR-106	MYL4
AF	300	1.98 ± 1.24	0.24 ± 0.08
Control	300	1.42 ± 0.75	1.55 ± 0.79
*t*		6.693	28.575
*P*		<0.001	<0.001

**Table 3:** Comparison of serum miR-106 and MYL4 levels in patients with different types of AF ($\bar{x}\pm s$).

Groups	Cases	miR-106	MYL4
Paroxysmal AF	124	1.54 ± 0.76^∗^	1.05 ± 0.65^∗^
Persistent AF	113	1.68 ± 0.85^∗^	0.75 ± 0.58^∗^
Long-term persistent AF	42	1.80 ± 0.97^∗^	0.46 ± 0.23^∗^
Permanent AF	21	2.01 ± 1.05^∗^	0.21 ± 0.18^∗^
Control	300	1.42 ± 0.75	1.55 ± 0.79
*F*		5.47	56.27
*P*		0.001	<0.001

**Table 4:** Prevalence of AF in patients with different levels of miR-106 and MYL4 [*n* (%)].

Groups		Cases	Morbidity
miR-106	Q1 < 1.10	150	33 (22.00)
	1.10 ≤ Q2 < 1.96	150	51 (34.00)
	1.96 ≤ Q3 < 2.82	151	88 (58.28)
	Q4 ≥ 2.82	149	128 (85.91)

MYL4	Q1 > 0.29	152	29 (19.08)
	0.29 ≥ Q2 > 0.24	149	58 (38.93)
	0.24 ≥ Q3 > 0.19	151	96 (63.58)
	Q4 ≤ 0.19	148	117 (79.05)

**Table 5:** Risk of AF prevalence with different serum miR-106 and MYL4 levels [*n* (%)].

Indicators	Quartile	Prevalence of AF (%)	OR value	95% CI value	*P* value
miR-106	Q1 (*n* = 150)	33 (22.00)	1.00	—	—
	Q2 (*n* = 150)	51 (34.00)	1.61	1.10~2.46	0.016
	Q3 (*n* = 151)	88 (58.28)	2.79	1.90~3.87	<0.001
	Q4 (*n* = 149)	128 (85.91)	4.08	2.88~5.74	<0.001

MYL4	Q1 (*n* = 152)	29 (19.08)	1.00	—	—
	Q2 (*n* = 149)	58 (38.93)	1.53	1.06~2.51	0.022
	Q3 (*n* = 151)	96 (63.58)	2.98	1.94~4.02	<0.001
	Q4 (*n* = 148)	117 (79.05)	4.52	2.25~4.98	<0.001

**Table 6:** Relationship between serum miR-106 and MYL4 levels and risk stratification of AF ($\bar{x}\pm s$).

CHA2DS2 score	Cases	miR-106	MYL4
0	15	1.12 ± 0.49	1.46 ± 0.87
1	64	1.55 ± 0.53	1.30 ± 0.67
2	71	1.63 ± 0.87	0.97 ± 0.46
3	92	1.72 ± 0.76	0.85 ± 0.14
4	33	1.87 ± 0.72	0.56 ± 0.13
5	14	1.95 ± 1.02	0.41 ± 0.24
6	11	2.05 ± 1.33	0.20 ± 0.09

**Table 7:** Differences between the indicators.

Factors	*P*	HR	95% CI
miR-106 ≤ 1.96 vs. miR-106 ≥ 1.96	<0.001	1.658	1.045~2.778
MYL4 ≥ 0.24 vs.MYL4 ≤ 0.24	<0.001	2.478	1.876~6.458
TC ≤ 4.5 mmol/L vs.TC > 4.5 mmol/L	<0.001	1.894	1.012~3.547
LDL-C ≤ 2.6 mmol/L vs. LDL-C > 2.6 mmol/L	<0.001	2.013	1.230~3.778

## Data Availability

The datasets used or analyzed during the current study are available from the corresponding author on reasonable request.
